# Cube-Related Corner Coalesced Nets

**DOI:** 10.3390/molecules24071221

**Published:** 2019-03-28

**Authors:** Mircea V. Diudea

**Affiliations:** Department of Chemistry, Faculty of Chemistry and Chemical Engineering, Babes-Bolyai University, Arany J. Street 11, Cluj 400028, Romania; diudea@gmail.com; Tel.: +40-745934281

**Keywords:** cube, *pcu*, *flu*, *dia*, spongy, diamondoid net, topology

## Abstract

Finite or periodic structures containing the cube motif can be synthesized and transformed into a variety of structures both at the theoretical and real, experimental level. The rhombellation topo-geometric operation may be used to transform the cube-shape into larger units and then build light (spongy) structures with larger voids. Hyper-clusters are polyhedral structures which nodes are polyhedral structures (the same or different ones). The paper presents some hypothetical spongy structures related to the cubic primitive *pcu*-net, with defects induced by cutting-off some atoms and/or bonds so that only corners are shared between two cubes. A diamondoid hyper-structure containing cube-coalesced corners was proposed for an eventual synthesis. The discussed structures are described in topological terms, particularly by sequential vertex connectivity and ring environment.

## 1. Introduction

Nanoscience is a general term used to specify the level of knowledge about structures with nanometer dimensions (1 nanometer = 10^−9^ m). Together with the technology enabling the application of nanoscience, in a variety of fields, including science, technique, health, society and culture (also termed nanotechnology), they collectively define the “nanoera” a new time of unprecedented progress in human life related to nanoscience [1]. Research for new materials with applications in technology or health care is an important task nowadays. The efforts are justified by the need for special properties, e.g., light and strong, biodegradable and low polluting materials and processes.

Out of the natural or synthesized materials like diamond, diamond-like carbon, metal oxides, etc., used for their high density/hardness or other physico-chemical properties, zeolites [2] and metal-organic frameworks (MOFs) [3,4,5] are appreciated for their light structures, with voids inside that can be occupied by appropriate guests, or remain empty. In this article, such light structures are termed “spongy” ones.

**Figure 1:**
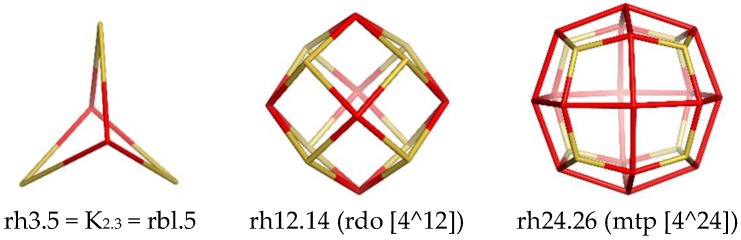
Rhomb tessellated units.

Rhombellanes are mathematical structures, introduced by us in 2017 [6]. A rhombellane was defined [7,8,9,10] as a structure with the following characteristics: (1) all strong rings are rhombs/squares; (2) its vertex class consists of all non-connected vertices; (3) the omega polynomial has a single term: 1X^|E(G)|^; (4) the line graph of the parent graph shows a Hamiltonian circuit; (5) it haa at least one rbl.5, the smallest rhombellane. Rombellanes originate from real molecules, such as propellanes [11] and polymeric staffanes [12]. The smallest rhombellane, rbl.5 is the realization of K_2,3_—a complete bipartite graph (Figure 1, left). By accepting larger even-sized polygonal faces (with respect to the Omega criterion) and tiles other than rbl.5, one may define related quasi-rhombellanes.

The aim of this paper is to introduce some spongy nets related to corner coalesced cubic nets and, on this basis, to propose possible synthesizable networks, candidates to biodegradable polymers.

## 2. Results

Cube-shape-containing structures can be modified by using topo-geometric operations, particularly the rhombellation operation. Out of the smallest rhombic shape, rh6.8 (i.e., the shape of cube–RCSR symbol [13]: cub [4^6])), other polyhedral shapes can be obtained, like those shown in Figure 1: rh12.14 (rdo [4^12]—middle) and rh24.26 (mtp [4^24]—right). These shapes result by iterating the rhombellation on the cube-shape-containing structures; the number of rhombs/squares in the actual generation, is twice that in the previous one. The number suffixing the name of a structure counts its vertices/atoms (Remark: in the above examples, this number is larger by 2 than the number of rhombs, a consequence of the Euler characteristic for the structures embedded in the sphere, by performing such operations, see [14]). Note that “shape” is used here instead of the corresponding polyhedron, since, in this topological view, angles and bond lengths are disregarded [15]. 

### 2.1. Cube-Shape Corner Coalesced Nets

**Figure 2:**
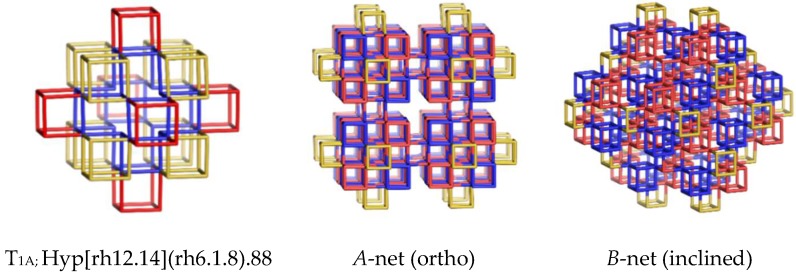
Corner-coalesced cube nets.

**Figure 3:**
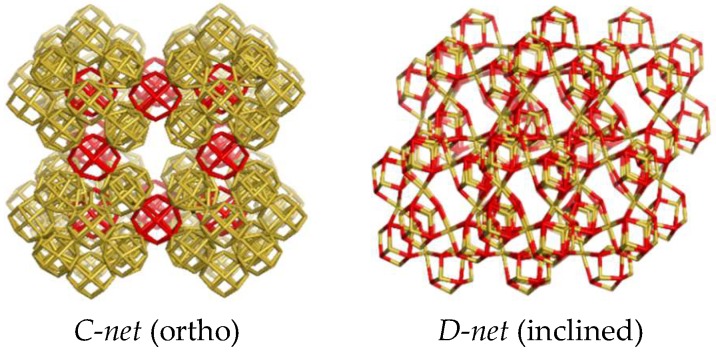
Rhombellated nets of Figure 2.

**Figure 4:**
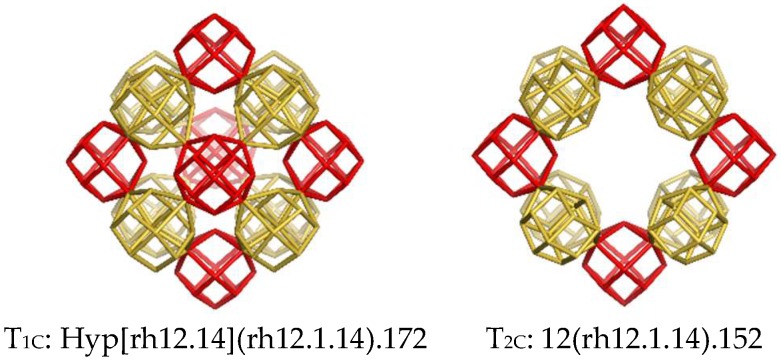
Tiles in the rhombellated nets of Figure 3.

The interest here was focused on finding structures made by constructing polyhedral shapes having coalesced corners and no edges or faces shared by two cells/shapes. In this respect, a domain consisting of 14 cubic shapes was cut off from the cubic primitive *pcu*-net. Its translation along the xyz axes led to two nets, denoted here as A and B (Figure 2, middle and right), where the B-net resulted from translating the domain/unit (T_1A_—Figure 2, left; Table 1, entry 1) by using an inclined (by 60°) z-coordinate [16,17]. Capital letters will be assigned for the hereafter discussed nets, except for the known ones. Rhombellation of these two nets led to the corresponding nets shown in Figure 3; the units of the rhombellated nets are shown in Figure 4 (see also Table 1, entries 3 and 4). 

The shapes making up these nets are indicated in Table 1 (fourth column), while the tiles (with subscript numbers and letters) are listed in the last column; tiles are repeating units, building blocks, etc. (for a more complete definition see [9,18,19]).

### 2.2. Spongy Corner Coalesced Nets

**Figure 5:**
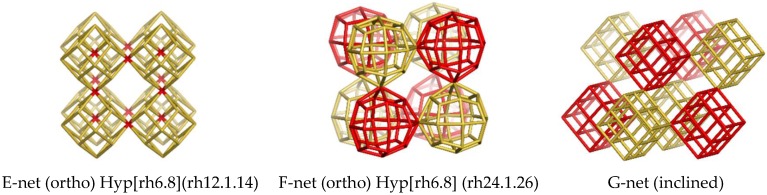
Corner-coalesced nets.

Other nets were built by using direct translation of appropriate units (Figure 5, left and right) or the rhombellation operation (Figure 5, middle). Details are found in Table 1, entries 5 to 7.

### 2.3. Hyper-Diamondoid Nets

**Figure 6:**
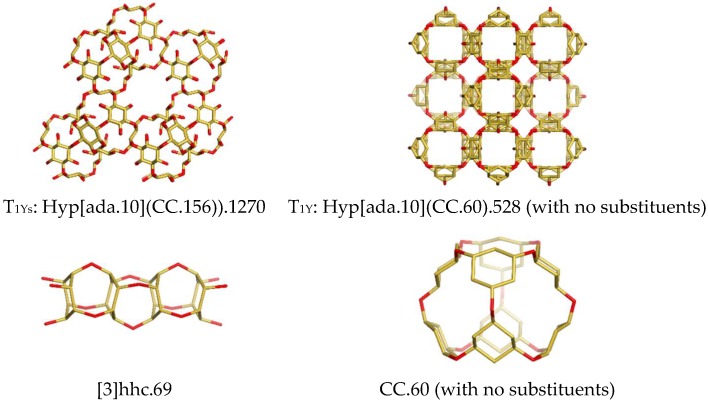
Hyper-adamantane (left, top), the unit of a triple periodic diamondoid net; a rod-like etheric relative (left, bottom) and the building blocks of the triple periodic net (with no substituents-the right column).

A triple periodic hyper-network, denoted here as Y-net was designed by using as the repeating unit a hyper-adamantane Hyp[ada.10](CC.156)).1270, (Figure 6, left, top); it is made from etheric cuboids CC.156, formally derived from hexahydroxycyclohexane, *hhc*. The suffixing numbers include here both the cyclohexane substituents and hydrogen atoms; for the sake of simplicity, these are further omitted, e.g., CC.156 becomes CC.60 (see Figure 6, the right column and Table 1, entry 9). A corresponding rod-like net is shown in Figure 6 (left, bottom). 

The topology of the structures above presented is basically characterized by sequences of connectivity (LC) and rings around a vertex (LR) [20,21,22]. The LR matrix provided different values for different ring domains, e.g., for (r_min_.r_min_) (corresponding to the ring symbol) and (r_min_.r), r-being a chosen value; these values are indicated in the tables listing LC and LR sequences (Appendix A, Table A1, Table A2, Table A3 and Table A4). 

## 3. Discussion

Crystals are highly ordered structures, with periodically repeating atomic clusters in three independent directions of space, and showing an essentially discrete diffraction diagram [23]; the symmetry of infinite crystal lattices is completely described by the 230 space symmetry groups.

### 3.1. Cube-Shape Containing Structures

Rhombellation operation applied to the *pcu*-net (tile rh6.8, i.e., the cube or better a cuboid shape) leads to a net where the shape has twice the number of rhombs, namely rh12.14. Iterating the operation, rbl(rbl(*pcu*)) results in a network with the shape rh24.26 [24]. 

Hyper-clusters are those polyhedral structures of which the nodes are polyhedral structures (the same or different ones). There are few convex polyhedra that are true fillers of 3D space [10], e.g., the cube, the rhombic dodecahedron, rdo, etc. Then, a hyper-cluster of rdo (better of its shape, rh12.14), like Hyp[rh12.14](rh6.1.8).88 (i.e., the tile T_1A_ of A- and B- nets), may be viewed as a filler of a “hyper-space”. The square brackets give information about the “host” cluster, of which vertices consist of the second cluster (round brackets). The name of shapes, being the “bricks” of the host cluster are eventually amended by the number of vertices/atoms shared by two shapes/cells in the realization of the hyper-cluster, e.g., “rh6.1.8” means corner-coalesced cubic shapes. The herein discussed hyper-tiles: T_1A_, T_1C_ and T_1Y_, are listed in Table 1 (entries 2, 4 and 9) and also shown in Figure 2, Figure 4 and Figure 6. The tile T_2A_: (rh6.e.8@6(rh6.8).32), is in fact a void; the letter “e” indicates the core, a “cube with no edges”.

### 3.2. Spongy Corner Coalesced Nets

The vertex/atom coalescence of cells is a fact well-known from fullerenes. If, in a net, there is one tile and one void (complementary ones), both of which can be used to generate the same net. The *flu*-net may be generated both by ortho and inclined units; there is a third way, using the same unit (i.e., rh12.14) to give the corner-coalesced E-net (Table 1, entry 5). The inclined coordinates lead to the same net in the case of simple units (see the *flu*-net) while different nets result when the units are more complex (see the B- and G- nets). The F-net (Table 1, entry 6) was designed by rhombellating the E-net. The sequences for the *flu*- and *pcu*-nets are given in Table A3.

In the both E- and F- nets one may cut-off domains of eight shapes/cells that can be viewed as hyper-structures: Hyp[rh6.8](rh12.1.14) (Figure 5, left); Hyp[rh6.8] (rh24.1.26) (Figure 5, middle). The inside void is the same as the corresponding shape; translating the E-net to fit with its voids, and identifying the superposed vertices, one obtains the well-known *flu*-net Table A3 (entry 2). In the case of the F-net, the “entanglement” with itself leads to a (spongy) *pcu*-net with defects (namely the shape rh24.9.26–Table A3, entry 3), the H-net (Table 1, entry 8). The design of the “half”-nets, E and F, of the *flu*- and *pcu*-(defect) nets, respectively, may be useful in understanding the structural details and relatedness of nets, apparently very different.

### 3.3. Spongy Diamond Nets

In a previous paper [17] we designed a spongy-diamond net, of which the hyper-unit is Hyp[ada.10](ada.10).100. In the actual paper, the topology of the new diamondoid hyper-net Y (Table 1, entry 9) was established on the simplified tile T_1Y_: Hyp[ada.10](CC.60).528 (for the sequences LC and LR see Table A4) but the most important is the possible real triple periodic network built by T_1Ys_: Hyp[ada.10](CC.156)).1270 and finally by the cuboid CC.156 shapes. A synthesis may start from 1,2,3,4,5,6-hexahydroxycyclohexane, *hhc*, that may form an ether with itself: if the molecule conformation is all-axial, the polyether will be a linear rod-like structure (see Figure 6, left, bottom) while in the all-equatorial conformation, the triple-periodic Y-net may be formed, by means of Hyp[ada.10](CC.156)).1270 and the cuboid shape CC.156. Energetic aspects, computed at the DFT level of theory, and pharmacological properties of a double-shell cluster built from CC.156, were published in a previous paper of Topo Group Cluj [25]. Substructures of this double-shell rhombellanic cuboid were tested for virtual docking with two indolizine derivatives, with good results [26]. Also, the linear hypothetical polymer, [n]hhc, (Figure 6, left, bottom) was docked to the enzyme glucose oxidase, GOX (3QVR) [27]. These results are a promise for the design and synthesis of cuboid-based molecules. In recent years, several research groups have reported hyper-structures, both as hypothetical and realized molecules [28,29,30,31,32,33,34,35,36]. For cubanes, see refs. [37,38,39]. The paper is an attempt to describe possible ways to access new, finite or periodic structures, in topological terms, rather than crystallographic ones. The relation with synthetic chemistry was established by the diamondoid Y-net.

## 4. Methods

Cube-shape containing structures were modified by using topo-geometric operations, particularly the rhombellation operation. In addition to the smallest rhombic shape, rh6.8 (i.e., the shape of cube), there appear other polyhedral shapes, like rh12.14, rh24.26, etc. These shapes result by iterating the rhombellation on the cube-shape-containing structures. Rhombellation [24] starts by diagonalizing each face of an all-rhomb map rh_0_ by a joint point (called “rbl-point”); then, new vertices are added, opposite to the parent vertices and joined, each of them, with the rbl-vertices lying in the proximity of a parent vertex, thus local rh-cells are formed. The process can be iterated, considering the envelope/shell rh*_n_* as “rh_0_” for rh_*n*+1_, and in this way shell by shell are added to the precedent structure. Since the two diagonals of a rhomb may be topologically different, each new generation may provide two isomers. Here, rhombellation was used to achieve the doubling of the number of rhombs in a cell, included in a periodic net. Finding the vertex (subgraph) classes in a graph is related to topological symmetry; they are calculated as centrality classes, by using the centrality index, C, developed at Topo Group Cluj [20]. It is calculated on layer/shell matrices [21,22], by the formula:

$$C{\left(\mathrm{LM}\backslash \mathrm{ShM}\right)}_{i}={\left[\sum_{k=1}^{ecc_{i}}{\left({\left[\mathrm{LM}\backslash \mathrm{ShM}\right]}_{ik}^{2k}\right)}^{1/{\left(ecc_{i}\right)}^{2}}\right]}^{-1};\;C(\mathrm{LM}\backslash \mathrm{ShM})=\sum_{i}C{\left(\mathrm{LM}\backslash \mathrm{ShM}\right)}_{i}$$

This index allows one to find the graph center and provides an ordering of vertices according to their centrality [38]. All computations have been using our original Nano Studio software package [39].

## 5. Conclusions

Atom coalescence of cells is a fact already known in fullerenes [40]. Structures like those discussed above may appear at the impact of mater with an ion beam, laser, etc., while the experimentalist needs models for structure identification and properties checking. Corner-coalesced structures can be viewed as light/spongy materials, related to zeolites or MOFs. Cuboid structures were modeled at the Topo Group Cluj in an attempt to design rhombellanic real molecules, of which pharmacological properties were simulated [25]. The six-connected vertex of a corner-coalesced cuboid may be obtained from the hexahydroxy-cyclohexane. The rod-like structure and the etheric triple periodic net, possible to be synthesized from the CC.156 cuboid, represent polymers that may undergo biodegradation in environment, if ever synthesized. The topology of the discussed structures was given in terms of substructure composition, atom connectivity LC and rings around RC sequences.

## Figures and Tables

**Table 1:** Names of structures, assumed letter, shape, symbol and tile topology.

#	Net Name	Letter	Shape (Symbol)	Tile (Topology)
1	Corner coalesced *pcu* (ortho)	A	rh6.8 (cub [4^6])	T_1A_: (14(rh6.1.8)); (v = 88; e = 168; r_4_ = 84; r_6_ = 48; r_10_ = 96; r_12_ = 998). T_2A_: (rh6.e.8@6(rh6.8)); (v = 32; e = 48; r_4_ = 6; r_6_ = 12).
2	Corner coalesced *pcu* (inclined)	B	rh6.8 (cub [4^6])	T_1A_: Hyp[rh12.14](rh6.1.8).88.
3	Rhombellated-A; rbl(A (ortho))	C	rh12.14 (rdo [4^12])	T_1C_: (14(rh12.1.14)); (v = 172; e = 336; r_4_ = 168; r_8_ = 300). T_2C_: 12(rh12.1.14); (v = 152; e = 288; r_4_ =1 44; r_8_ = 232).
4	Rhombellated-B; rbl(B (inclined))	D	rh12.14 (rdo [4^12])	T_1C_: Hyp[rh12.14](rh12.1.14).172
5	Corner coalesced *flu* (spongy; ortho)	E	rh12.14 (rdo [4^12])	T_1E_: (rh12.1.14); (v = 14 ([6(4^4).8(4^3)]); e = 24; r_4_ = 12; r_8_ = 18). T_2E_: Void = T_1E_.
6	Rhombellated-E; rbl(E (spongy; ortho))	F	rh24.26 (mtp [4^24])	T_1F_: (rh24.1.26); (v = 26 ([6(4^4).12(4^4).8(4^3)]); e = 48; r_4_ = 24; r_8_ = 15; r_10_ = 109). T_2F_: Void = T_1F_.
7	Corner coalesced *pcu* (spongy; inclined)	G	rh24.26 (mtp [4^24])	T_1F_; T_2F_
8	Deffect (mtp) *pcu* (spongy; ortho)	H	rh24.26 (mtp [4^24])	T_1H_: (rh24.9.26).
9	Etheric hyper-diamond	Y	CC.60	T_1Y_: (10CC.60); (v = 528; e = 648; r_6_ = 68; r_16_ = 60). CC.60 (v = 60; e = 72; r_6_ = 8; r_16_ = 6). T_1Y_: Hyp[ada.10](CC.60).528T_1Ys_: Hyp[ada.10](CC.156)).1270

## Appendix

**Table A1:** Corner coalesced cubic *pcu* network: unit/tile; vertex connectivity classes; ring domain; population; degree; point symbol; LM sequence: connectivity (LC) and atom surrounding rings (LR).

Cn Cls	Network Letter and Vertex Classes	LM
1	**A (ortho)**	T_1_: (v = 88; e = 168; r_4_ = 84; r_6_ = 48; r_10_ = 96; r_12_ = 998)
1.1	deg = 3; {40}; 4^3	
1.1.1	(4.4); {8}; deg = 3; 4^3 (4.6); {8}; deg = 3; 4^3	LC: {8}; 3.3.10.30.45.54.90.145.169.196. LR(4.4): {8}; 3.9.18.57.117.171.261.405.552.696.921. LR(4.6); {8}; 3.15.42.135.255.357.561.885.1170.1452.1983.
1.1.2	(4.4); {24}; deg = 3; 4^3 (4.6); {24}; deg = 3; 4^3.6^2	LC: {24}; 3.9.23.35.47.82.127.153.194.277. LR(4.4): {24}; 3.15.45.90.141.225.354.495.657.885.1137. LR(4.6); {24}; 5.31.103.198.297.487.772.1047.1385.1907.2427.
1.1.3	(4.4); {8}; deg = 3; 4^3 (4.6); {8}; deg = 3; 4^3.6^6	LC: {8}; 3.12.31.45.54.90.145.169.196.286. LR(4.4): {8}; 3.18.63.120.171.261.405.552.696.921.1218. LR(4.6): {8}; 9.42.141.258.357.561.885.1170.1452.1983.2628.
1.2	deg = 6; {48}; 4^6	
1.2.1	{4.4}; {48}; deg = 6; 4^6 {4.6}; {48}; deg = 6; 4^6.6^8	LC: {48}; 6.15.24.39.68.98.126.177.242.283. LR(4.4): {48}; 6.27.60.105.177.279.402.558.762.975.1161. LR(4.6); {48}; 14.61.130.225.385.601.852.1192.1636.2063.2437.
2	**B (inclined)**	T_1_: (v = 88; e = 168; r_4_ = 84; r_6_ = 48; r_10_ = 96; r_12_ = 998)
2.1	deg = 3; {32}; 4^3	
2.1.1	(4.4); {32}; deg = 3; 4^3 (4.6); {32}; deg = 3; 4^3.6^6	LC: {32}; 3.12.31.45.60.109.166.181.205.306. LR(4.4): {32}; 3.18.63.123.189.324.519.660.789.1056.1413. LR(4.6): {32}; 9.54.189.369.567.972.1557.1980.2367.3168.4239.
2.2	deg = 6; {56}; 4^6	
2.2.1	(4.4); {56}; deg = 6; 4^6 (4.6); {56}; deg = 6; 4^6.6^12	LC: {56}; 6.15.26.48.84.112.135.190.262.303. LR(4.4): {56}; 6.27.63.126.234.360.480.648.894.1131.1323. LR(4.6): {56}; 18.81.189.378.702.1080.1440.1944.2682.3393.3965.
3	**C (ortho)**	T_1_: 14(12.1.14); (v = 172; e = 336; r_4_ = 168; r_8_ = 300); T_2_: 12(12.1.14); (v = 152; e = 288; r_4_ = 144; r_8_ = 232).
3.1	deg = 3; {40}; 4^3	
3.1.1	(4.4); {16}; deg = 3; 4^3 (4.8); {8}; deg = 3; 4^3.8^9 (4.8); {8}; deg = 3; 4^3.8^9	LC: {16}; 3.6.12.19.60.93.105.111.264.374. LR(4.4): {16}; 3.12.27.48.111.240.369.420.594.1056.1446. LR(4.8): {8}; 12.48.132.252.588.1140.1716.2040.3072.4992.6648. LR(4.8): {8}; 12.60.132.240.588.1140.1716.2040.3072.4992.6648.
3.1.2	(4.4); {24}; deg = 3; 4^3 (4.8); {24}; deg = 3; 4^3.8^9	LC: {24}; 3.6.9.13.44.72.93.106.224.315. LR(4.4): {24}; 3.12.24.36.78.176.297.372.534.896.1263. LR(4.8): {24}; 12.52.112.184.416.844.1404.1800.2712.4252.5900.
3.2	deg = 4; {84}; 4^4	
3.2.1	(4.4); {24}; deg = 4; 4^4 (4.8); {24}; deg = 4; 4^4.8^12	LC: {24}; 4.10.16.45.72.93.106.224.315.337. LR(4.4): {24}; 4.15.28.54.112.210.312.459.688.984.1236. LR(4.8): {24}; 16.68.136.280.548.1024.1500.2260.3288.4728.5912.
3.2.2	(4.4); {24}; deg = 4; 4^4 (4.8); {24}; deg = 4; 4^4.8^16	LC: {24}; 4.7.10.28.47.78.100.172.229.309. LR(4.4): {24}; 4.18.40.87.180.297.372.534.896.1263.1348. LR(4.8): {24}; 20.88.200.452.860.1404.1800.2712.4252.5900.6492.
3.2.3	(4.4); {24}; deg = 4; 4^4 (4.8); {24}; deg = 4; 4^4.8^16	LC: {24}; 4.16.28.45.60.128.184.205.216.442. LR(4.4): {24}; 4.24.64.120.180.300.512.744.820.1080.1768. LR(4.8): {24}; 20.128.312.576.876.1520.2432.3488.3956.5472.8376.
3.2.4	(4.4); {24}; deg = 4; 4^4 (4.8); {12}; deg = 4; 4^4.8^20	LC: {12}; 4.16.28.41.52.104.148.197.228.390. LR(4.4): {12}; 4.24.64.120.164.252.416.624.788.1068.1560. LR(4.8): {12}; 24.128.304.576.792.1264.1984.2976.3784.5296.7408.
3.3	deg = 6; {48}; 4^6	
3.3.1	(4.4); {48}; deg = 6; 4^6 (4.8); {48}; deg = 6; 4^6.8^26	LC: {48}; 6.12.30.48.78.100.172.229.309.352. LR(4.4): {48}; 6.24.60.120.213.312.459.688.984.1236.1578. LR(4.8): {48}; 32.120.304.580.1036.1500.2260.3288.4728.5912.7704.
4	**D (inclined)**	T_1_: 14(12.1.14); (v = 172; e = 336; r_4_ = 168; r_8_ = 300).
4.1	deg = 3; {32}; 4^3	
4.1.1	(4.4); {32}; deg = 3; 4^3 (4.8); {32}; deg = 3; 4^3.8^9	LC: {32}; 3.6.12.19.63.96.141.154.345.410. LR(4.4): {32}; 3.12.27.48.114.252.432.564.834.1380.1746. LR(4.8): {32};12.60.144.276.684.1308.2304.3156.4824.7116.9048.
4.2	deg = 4; {84}; 4^4	
4.2.1	(4.4); {48}; deg = 4; 4^4 (4.8); {48}; deg = 4; 4^4.8^16	LC: {48}; 4.10.16.48.76.126.146.304.360.420. LR(4.4): {48}; 4.18.40.90.192.354.504.762.1216.1578.1680. LR(4.8): {48}; 20.96.224.528.1008.1920.2784.4344.6336.8304.9280.
4.2.2	(4.4); {36}; deg = 4; 4^4 (4.8); {36}; deg = 4; 4^4.8^20	LC: {36}; 4.16.28.53.72.168.216.273.272.542. LR(4.4): {36}; 4.24.64.132.212.384.672.960.1092.1416.2168. LR(4.8): {36}; 24.144.336.720.1176.2208.3504.5088.6040.8064.11248.
4.3	deg = 6; {56}; 4^6	
4.3.1	(4.4); {56}; deg = 6; 4^6 (4.8); {56}; deg = 6; 4^6.8^30	LC: {56}: 6.12.33.53.105.132.240.277.399.407. LR(4.4): {56}; 6.24.63.132.261.420.648.960.1290.1596.1926. LR(4.8): {56}; 36.132.360.708.1452.2268.3600.5112.6996.8580.10524.

**Table A2:** Spongy corner coalesced networks: unit/tile; vertex connectivity classes; ring domain; population; degree; point symbol; LM sequence: connectivity (LC) and atom surrounding rings (LR).

Cn Cls	Network Letter and Vertex Classes	LM
1	**E (ortho)**	T_1_: (rh12.1.14); (v = 14 ([6(4^4).8(4^3)]); e = 24; r_4_ = 12; r_8_ = 18). T_2_: Void = T_1_.
1.1	deg = 3; {8}; 4^3	
	(4.4); {8}; deg = 3; 4^3 (4.8); {8}; deg = 3; 4^3.8^33	LC: {8}; 3.18.15.67.45.166.90.305.150.478. LR(4.4): {8}; 3.24.54.120.201.360.498.720.915.1200.1434. LR(4.8): {8}; 36.288.648.1440.2412.4320.5976.8640.10980.14400.17208.
1.2	deg = 8; {8}; 4^8	
	(4.4); {6}; deg = 8; 4^8 (4.8); {6}; deg = 8; 4^8.8^88	LC: {6}; 8.8.40.30.120.68.240.126.400.180. LR(4.4): {6}; 8.24.64.120.240.360.544.720.1008.1200.1440. LR(4.8): {6}; 96.288.768.1440.2880.4320.6528.8640.12096.14400.17280.
2	**F (ortho)**	T_1_: (rh24.1.26); (v = 26 ([6(4^4).12(4^4).8(4^3)]); e = 48; r_4_ = 24; r_8_ = 15; r_10_ = 109). T_2_: Void = T_1_.
2.1	deg = 3; {8}; 4^3	
2.1.1	(4.4); {8}; deg = 3; 4^3 (4.8); {8}; deg = 3; 4^3	LC: {8}; 3.6.18.30.84.112.225.256.420.455. LR(4.4): {8}; 3.12.33.72.165.336.561.900.1218.1680.2115. LR(4.8): {8}; 3.30.105.180.525.840.1641.2250.3378.4200.5715.
2.2	deg = 4; {12}; 4^4	
2.2.1	(4.4); {12}; deg = 4; 4^4 (4.8); {12}; deg = 4; 4^4.8^6	LC: {12}; 4.14.24.68.96.206.230.372.426.670. LR(4.4): {12}; 4.22.56.132.272.488.824.1090.1488.2008.2680. LR(4.8): {12}; 10.70.140.420.680.1448.2060.3010.3720.5512.6700.
2.3	deg = 8; {6}; 4^8	
2.3.1	(4.4); {6}; deg = 8; 4^8 (4.8); {6}; deg = 8; 4^8.8^24	LC: {6}; 8.16.48.70.160.188.320.366.584.580. LR(4.4): {6}; 8.32.88.192.360.640.904.1280.1728.2336.2640. LR(4.8): {6}; 32.80.280.480.1080.1600.2536.3200.4752.5840.6960.
3	**G (inclined)**	T_1_: (rh24.1.26); (v = 26 ([6(4^4).12(4^4).8(4^3)]); e = 48; r_4_ = 24; r_8_ = 15; r_10_ = 109). T_2_: Void = T_1_.
3.1	(4.4); 6 | deg = 4 | 4^4 (4.8); 6 | deg = 4 | 4^4.8^10	LC: {6}; 4.8.20.29.64.81.140.154.236.242. LR(4.4): {6}; 4.16.40.80.148.256.396.560.744.944.1168. LR(4.8): {6}; 14.44.104.220.374.704.1062.1540.2028.2596.3188.
3.2	(4.4); 12 | deg = 4 | 4^4 (4.8); 12 | deg = 4 | 4^4.8^7	LC: {12}; 4.12.20.45.58.105.120.190.200.298LR(4.4): {12}; 4.20.48.100.180.284.420.580.760.964.1192. LR(4.8): {12}; 11.52.132.260.495.760.1155.1580.2090.2636.3278.
3.3	(4.4); 8 | deg = 6 | 4^6 (4.8); 8 | deg = 6 | 4^6.8^6	LC: {8}; 6.12.30.42.78.92.150.162.246.252. LR(4.4): {8}; 6.24.60.120.204.312.444.600.780.984.1212. LR(4.8): {8}; 12.66.156.330.552.858.1212.1650.2136.2706.3306.

**Table A3:** Corner coalesced *pcu*, *flu* and deffect (mtp) *pcu* networks: unit/tile; vertex connectivity classes; ring domain; population; degree; point symbol; LM sequence: connectivity (LC) and atom surrounding rings (LR).

Cn Cls	Network Letter and Vertex Classes	LM
1	***pcu* (ortho)**	T_1_: (v = 8; e = 12; r_4_ = 6)
1.1	deg = 6; {8}; 4^12	
1.1.1	(4.4); {8}; deg = 6; 4^12	LC: {8}; 6.18.38.63.84.92.84.63.38–18 LR(4.4): {8}; 12.72.216.444.696.864.867.708.465.240.93
2	***flu* (ortho)**	T_1_: (12.4.14); (v = 14([6(4^4).8(4^3)]); e = 24; (r_4_ = 12; r_8_ = 18).
	deg = 4; {8}; 4^6	
2.1	(4.4); {8}; deg = 4; 4^6 (4.8); {8}; deg = 4; 4^6.8^36	LC: {8}; 4.22.24.82.64.182.124.322.204.502.304. LR(4.4): {8}; 6.48.132.288.492.768.1092.1488.1932.2448.3012.3648. LR(4.8): {8}; 42.336.924.2016.3444.5376.7644.10416.13524.17136.21084.
2.2	(4.4); {6}; deg = 8; 4^12 (4.8); {6}; deg = 8; 4^12.8^72	LC: {6}; 8.12.48.42.128.92.248.162.408.252.608. LR(4.4): {6}; 12.48.144.288.504.768.1104.1488.1944.2448.3024.3648. LR(4.8): {6}; 84.336.1008.2016.3528.5376.7728.10416.13608.17136.21168.
3	**H (ortho)**	T_1H_: (rh24.9.26). (v = 26 ([6(4^4).12(4^4).8(4^3)]); e = 48; r_4_ = 24; r_8_ = 15; r_10_ = 109).
3.1	deg = 4; {6}; 4^4	
	(4.4); {6}; deg = 4; 4^4 (4.8); {6}; deg = 4; 4^4.8^20	LC: {6}; 4.16.28.66.76.146.148.258.244.402. LR(4.4): {6}; 4.32.96.224.392.608.872.1184.1544.1952.2408. LR(4.8): {6}; 24.112.336.784.1392.2128.3072.4144.5424.6832.8448.
	deg = 6; {8}; 4^12	
3.2	(4.4); {8}; deg = 6; 4^12	LC: {8}; 6.18.30.66.78.146.150.258.246.402.
	(4.8); {8}; deg = 6; 4^12	LR(4.4): {8}; 12.48.120.240.408.624.888.1200.1560.1968.2424.
		LR(4.8): {8}; 12.168.360.840.1368.2184.3048.4200.5400.6888.8424.
3.3	deg = 6; {12}; 4^8	
	(4.4); {12}; deg = 6; 4^8	LC: {12}; 6.14.38.50.102.110.198.194.326.302.
	(4.8); {12}; deg = 6; 4^8.8^20	LR(4.4): {12}; 8.40.112.232.400.616.880.1192.1552.1960.2416.
		LR(4.8): {12}; 28.120.392.792.1400.2136.3080.4152.5432.6840.8456.

**Table A4:** Etheric hyper-diamond (no substituents) network: unit/tile; vertex connectivity classes; ring domain; population; degree; point symbol; LM sequence: connectivity (LC) and atom surrounding rings (LR).

Cn Cls	Network Letter and Vertex Classes	LM
1	Y (hyper-ada)	T_1_: (v = 528; e = 648; r_6_ = 68; r_16_ = 60).
1.1	(6.16); {120}; deg = 2; 16^2; (120 free)	LC: {120}; 2.4.3.5.9.12.18.22.26.22. LR: {120}; 2.6.8.11.18.28.36.44.58.57.69.
1.2	(6.16); {120}; deg = 2; 6.16; (168 free)	LC: {120}; 2.4.6.8.9.13.20.20.24.28. LR: {120}; 2.7.12.18.20.24.39.52.58.68.82.
1.3	(6.16); {120}; deg = 3; 6.16^2; (168 free)	LC: {120}; 3.3.5.6.9.15.19.24.23.29. LR: {120}; 3.6.10.14.20.30.40.51.54.66.77.
1.4	(6.16); {168}; deg = 3; 6.16^3; (72 free)	LC:{168}; 3.5.7.9.12.14.16.20.23.28. LR:{168}; 4.10.15.18.22.29.36.50.66.70.71.
